# Mirror exposure therapies: Effect of the distance to the mirror on the attentional pattern in a Virtual Reality immersive environment

**DOI:** 10.1192/j.eurpsy.2023.907

**Published:** 2023-07-19

**Authors:** F.-A. Meschberger-Annweiler, M. Ascione, B. Porras-Garcia, H. Miquel, E. Exposito, E. Serrano-Troncoso, M. Carulla-Roig, M. Ferrer-Garcia, J. Gutierrez-Maldonado

**Affiliations:** 1 Department of Clinical Psychology and Psychobiology, University of Barcelona, Barcelona, Spain; 2 Department of Population Health Science, University of Utah School of Medicine, Salt Lake City, United States; 3 Child and Adolescent Psychiatry and Psychology Department, Hospital Sant Joan de Déu of Barcelona, Esplugues de Llobregat, Spain

## Abstract

**Introduction:**

Mirror exposure therapies (MET) have been proposed to reduce symptomatology in patients with Anorexia Nervosa. However, most MET protocols or related studies do not specify the patients’ distance to the mirror, or when they do so, such a distance may differ significantly (from 0,5 to 3 meters). Such modifications of mirror positioning could imply variations in patients’ fixation patterns on different body parts (i.e., attentional bias between weight-related and non-weight related body parts), since previous studies shown that dissociated neural systems (either in left or right cerebral hemispheres) are involved in the attentional patterns and scanning strategies depending on the distance (i.e., in near and far space). Furthermore, as the body-related attentional bias (AB) has been shown to be a part of the maintenance mechanism of AN symptomatology, any modification of attentional patterns due to mirror’s distance variations may influence the efficacy of MET.

**Objectives:**

This study aims to use Virtual Reality (VR) and Eye-Tracking (ET) technologies to precisely analyse the effect of the distance to the mirror on the attentional patterns.

**Methods:**

137 female college students were immersed in a VR environment in which they could look in the mirror at their respective avatars created from the measurements and photos of their real bodies. The mirror was positioned at 3.30m in front of the participants in “group 1” (n_1_ = 54), and at 1.54m in front of the participants in “group 2” (n_2_ = 83). Eye-Tracking feature and OGAMA software (Freie Universität, Berlin, Germany) were used to record and process the visual attentional pattern of each participant, during a 30-second free viewing task at her avatar. Complete Fixation Time (CFT) was assessed as the fixation time difference between weight- and non-weight- related body parts, defined from the weight scale of the PASTAS questionnaire. Independent Sample t-Test was conducted to analyse CFT mean difference between both groups.

**Results:**

Independent Samples t-Test shows statistically significant CFT mean difference (F (1, 135) = 1.571, p < 0.001, 95% IC [1717; 5581]) between both groups. While fixation pattern of the group positioned further to the mirror (group 1) was more focused on weight-related body parts (CFT mean = 2282ms, SD = 809), the fixation pattern of the group positioned closer to the mirror (group 2) was more focused on non-weight-related body parts (CFT mean = -1367ms, SD = 587).

**Conclusions:**

This study shows new opportunities to use VR and ET technologies to precisely analyse the variations of fixation patterns as a function of mirror position in MET. Such information may contribute to adapt and develop new MET’s protocols for AN patients, optimizing the distance to the mirror. It also underscores the importance of specifying the distance to the mirror in MET-related studies to improve replicability.

**Disclosure of Interest:**

None Declared

